# A Self-Supported Coral-like Pt/SnO_2_/Nb_2_O_5_/Nb Electrode: A Bifunctional Electrocatalyst for Membrane-Free Green H_2_ Production via Coupling Ethanol Selective Electrooxidation

**DOI:** 10.1021/acsomega.5c01734

**Published:** 2025-05-29

**Authors:** Matheus Bullmann, Andrés Cuña Suárez, Edna Jerusa Pacheco Sampaio, Natalia Prieto Pastorino, Roberto Hubler, Eva Chinarro, Célia de Fraga Malfatti

**Affiliations:** † Corrosion Research Laboratory (LAPEC), Engineering School, 28124 Federal University of Rio Grande do Sul (UFRGS), Av. Bento Gonçalves, 9500, 91501-970 Porto Alegre, Rio Grande do Sul, Brazil; ‡ Physical Chemistry Area, DETEMA, Faculty of Chemistry, University of the Republic (UDELAR), CC 1157, 11800 Montevideo, Uruguay; § Renewable Energies Laboratory, Pando Technological Pole Institute, Faculty of Chemistry, 201894 University of the Republic (UDELAR), Route 8 Km 17.500, 91000 Pando, Canelones, Uruguay; ∥ Polytechnic School, 28102 Pontifical Catholic University of Rio Grande do Sul (PUC-RS), Av. Ipiranga, 6681, 90619-900 Porto Alegre, Rio Grande do Sul, Brazil; ⊥ Institute of Ceramics and Glass (ICV), Higher Council for Scientific Research (CSIC), Cantoblanco Campus, C/Kelsen 5, 28049 Madrid, Spain

## Abstract

The production of green hydrogen via water electrolysis faces challenges due to the sluggish oxygen evolution reaction at the anode. In this study, we developed a self-supported bifunctional Pt/SnO_2_/Nb_2_O_5_/Nb electrocatalyst capable of coupling the hydrogen evolution reaction to the selective ethanol oxidation reaction (EOR) in an acidic medium. The catalyst demonstrated efficacy for the EOR, with an *E*_onset_ of +130 mV versus SHE results in a peak current density of 8.97 mA cm^–2^. The bifunctional electrocatalyst exhibited a spontaneous and rapid increase in current for the HER, achieving an overpotential of −48 mV for a current density of 5 mA cm^–2^ and a Faradaic efficiency of 99.7%. This efficient coupling of the EOR with the HER reduces energy consumption for green hydrogen generation compared to traditional water splitting. With hydrogen being the sole gaseous product, the HER–EOR electrolysis system can operate effectively without a membrane, promising a more cost-effective, sustainable, and efficient green hydrogen production.

## Graphical abstract

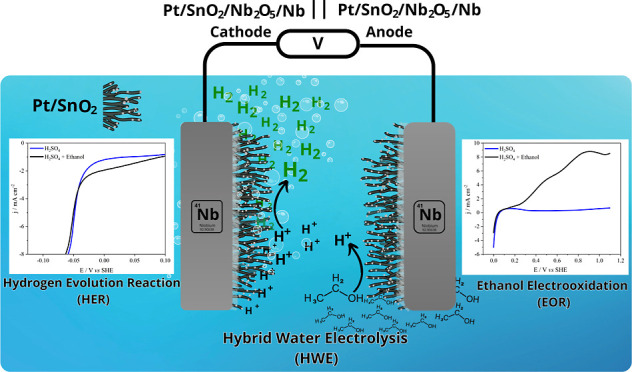

## 1 Introduction

The electrocatalytic production of green hydrogen has gained increasing attention in the technological scene due to the growing demand for energy and the rapid exhaustion of fossil fuels.^1,2^ Hydrogen gas (H_2_) is an abundant, environmentally friendly, and cost-effective energy source, boasting a higher energy density than gasoline and coal. Widely regarded as an ideal alternative to fossil fuels in the future, current global H_2_ production heavily depends on nonrenewable energy source processing, such as steam reforming, releasing substantial amounts of CO_2_ and contributing to global warming and unsustainable climate change.^3,4^

One of the most promising approaches for the efficient and environmentally friendly production of green H_2_ involves electrochemical water splitting (EWS) using proton exchange membrane (PEM) technology, which utilizes electricity from renewable sources such as wind, solar, and hydroelectric power.^5^ In this electrolysis process, water undergoes oxidation at the anode via the oxygen evolution reaction (OER) with a potential of 1.23 V versus the standard hydrogen electrode (SHE), generating H^+^ ions. These ions then migrate through the PEM to the cathode, where they undergo a reduction to H_2_ in the hydrogen evolution reaction (HER) at a standard potential of 0 V versus SHE. Thus, under standard conditions, the minimum thermodynamic voltage required for water decomposition to produce H_2_ is 1.23 V versus SHE, resulting in an extremely high level of energy consumption.^6,7^ Furthermore, in water electrolysis, the OER is typically limited by sluggish kinetics.^8,9^ When mixed with H_2_, oxygen forms an explosive H_2_/O_2_ mixture, posing potential safety hazards.^10^

In the EWS, the PEM facilitates cation transport, particularly hydrogen ions (H^+^), from the anodic to the cathodic compartment within the electrolytic cell. The PEM serves as a barrier between these compartments, ensuring selective ion transport while preventing the passage of electrons, and enables the efficient separation of hydrogen and oxygen gases produced during electrolysis. However, the implementation of expensive PEM in multiple devices is hindered by factors such as significant costs, limited availability in production, and restrictions on dimensions and mass.^11,12^

On the other hand, the hybrid water splitting approach utilizes the electrooxidation of organic substances, including alcohols,^13–17^ ammonia,^18,19^ and derivatives of biomass,^20,21^ in aqueous environments to generate hydrogen and produce valuable byproducts. These electrochemical reactions can take place at ambient temperatures and require low activation potentials.^22^ Additionally, this method circumvents the generation of oxygen gas, facilitating the creation of devices that function effectively without the need for a proton exchange membrane (PEM).

In conventional electrolyzers, the cathode and anode are typically made of different materials, which can cause cross-contamination and reduce electrode lifetime.^23^ Thus, the development of electrodes with selectivity for both reduction and oxidation enables bifunctional operation, allowing the same material to be used for both the cathode and the anode. This simplifies the system and can extend the lifetime of catalytic materials.^22^

Research is underway to explore hybrid water splitting. Zhou et al.^24^ presented a single-atom Ru-anchored porous Pt_3_Ni alloy (Ru_1_-Pt_3_Ni/NiF) as a bifunctional electrocatalyst for the concurrent ethanol oxidation reaction (EOR) and hydrogen evolution reaction (HER).

In a membrane-free system, the catalyst achieved a Faraday efficiency of 94% for H_2_ production with an energy consumption of 19.24 kW h per kg of H_2_. Zhang et al.^25^ demonstrated a Ni/MoC@NC electrocatalyst with bifunctional activity for the urea oxidation reaction (UOR) and HER. In a 0.5 M H_2_SO_4_ electrolyte, the catalyst achieved a current density of 10 mA cm^–2^ at an overpotential of 111 mV. Additionally, for urea electrolysis (HER||UOR), a cell voltage of 1.372 V was sufficient to reach 10 mA cm^–2^.

Noble metals supported on carbon, such as Pt,^26–28^ Pd,^29–32^ Au,^33,34^ and Ru,^35^ are employed in the EOR due to their catalytic characteristics and specific selectivity for this reaction. However, CO-like intermediates formed during the EOR can poison the active sites of noble metal catalysts, reducing the efficiency of the reaction.^36^

According to Fu et al.,^37^ introducing other elements to form Pt-based compound materials is a promising method to improve their EOR and HER activities. The addition of a cocatalyst, such as SnO_2_, creates a new catalytic pathway, enhancing selectivity and preserving the active sites of the metal noble catalyst for improved reaction efficiency.^38,39^

Kim et al.^40^ demonstrated that applying a SnO_2_ film to a carbon nanofiber (CNF) substrate significantly enhanced the dispersion of platinum particles on the support. XPS analysis further revealed an increased intensity of Pt spectra on the SnO_2_-coated CNF, highlighting an improved interaction between the catalyst and the ceramic support. Cyclic voltammetry studies confirmed that the island-shaped SnO_2_ coating on the CNF exhibited the highest current density performance, making it the most effective configuration for application in direct methanol fuel cells (DMFCs).

In traditional catalyst preparation methods, noble metal electrodes depend on carbon as support, current collectors, and binders like Nafion or polytetrafluoroethylene (PTFE).^41^ However, these methods require additional steps and effort to achieve a uniform nanoparticle dispersion.^42^

Research optimizing hydrogen production systems that depend on membrane devices in traditional water electrolysis is crucial for reducing costs and advancing this technology.^43^

Our study focuses on the development of a novel material in which the cocatalyst (SnO_2_) grows directly on a niobium substrate, known for its electrical conductivity and resistance to harsh conditions, forming self-supported electrodes. This design makes niobium a relevant metallic alternative for current collectors and supports for electrocatalysis.

This innovative approach eliminates the need for binders and augments the surface area through a semiconductor coral-like structure, synthesized by plasma electrolytic oxidation (PEO). When decorated with Pt via solvothermal synthesis, this material promotes the EOR in an acid medium with remarkable selectivity in C–C bond cleavage, as demonstrated in a previously published work by our research group.^38^

In this study, we successfully coupled the highly selective EOR with the HER, showcasing a Pt/SnO_2_/Nb_2_O_5_/Nb coral-like self-supported bifunctional electrocatalyst for use in membrane-free electrolyzers. Through electrochemical and chromatography tests, we demonstrate the efficient production of pure hydrogen via the electrochemical oxidation of ethanol under acidic conditions and at low potentials, offering a promising approach for the sustainable generation of hydrogen through a biomass precursor.

## 2 Experimental Section

### 2.1 Synthesis and Characterization of the Electrocatalyst

The synthesis and characterization of the Pt/SnO_2_/Nb_2_O_5_/Nb catalyst was performed and reported by Bullmann et al.^38^

In summary, metallic square samples of 1 cm × 1 cm were obtained from cutting a pure niobium plate. The surface of the metal samples was sanded up to 1200 grit and cleaned with an air plasma process of 250 μatm for 1 min. After that, the coral-like oxide structure (SnO_2_/Nb_2_O_5_/Nb) was synthesized via PEO. For the synthesis of the electrocatalysts (Pt/SnO_2_/Nb_2_O_5_/Nb), samples synthesized by PEO were decorated with platinum via the solvothermal process.

Scheme 1 illustrates the process of synthesis of Pt/SnO_2_/Nb_2_O_5_/Nb electrocatalysts for ethanol electrooxidation, which is utilized in producing hydrogen fuel and valuable chemicals.

**Scheme 1:**
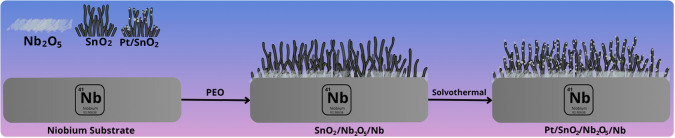
Schematic Illustration of the Preparation of Pt/SnO_2_/Nb_2_O_5_/Nb Electrocatalysts

Surface images were obtained through a JEOL JSM-7800F-Prime FEG model by scanning electron microscopy (SEM). The elemental composition of the samples was studied using a scanning electron microscope, Hitachi S-4700, coupled with an energy-dispersive spectroscopy (EDS) device.

The grazing incidence X-ray diffraction analysis (GIXRD) was performed using a Bruker D8 ADVANCE diffractometer (Bruker, Germany) (Cu Kα incident radiation, λ = 0.15418 nm), and measurements were conducted with an incidence angle of 1.5°. The GIXRD patterns were recorded with a step size of 0.02° over the range of 10–80°. X-ray diffraction (XRD) measurements were conducted before and after the stability tests using Cu Kα radiation over a 10–80° range at a scanning rate of 0.05° s^–1^.

The concentration of platinum in the electrocatalysts was determined by inductively coupled plasma optical emission spectrometry (ICP-OES). To extract Pt, the samples were digested in a 50:50 aqua regia solution under stirring and heating for 24 h. The resulting solution was then analyzed. Additionally, ICP-OES was employed to evaluate the leaching of metal ions into the electrolyte after stability tests.

### 2.2 Electrochemical Measurements

#### 2.2.1 Three-Electrode Configuration—Electrocatalyst Evaluation

To study the electrocatalytic performance of the Pt/SnO_2_/Nb_2_O_5_/Nb catalyst, different electrochemical measurements were performed using the potentiostat/galvanostat model Autolab PGSTAT 302 N at 25 °C. The electrochemical tests were performed in an electrochemical cell consisting of three electrodes, with platinum as the counter electrode, saturated Ag/AgCl as the reference electrode, and the self-supported electrocatalyst with coral-like structures (Pt/SnO_2_/Nb_2_O_5_/Nb) as the working electrode. The electrode used had a geometric area of 0.63 cm^2^.

To analyze the HER coupled with the EOR in acidic medium, linear voltammetry analyses were performed in a potential window of 0.0 V to −0.5 V versus saturated Ag/AgCl for the HER and −0.2 V to +0.9 V versus saturated Ag/AgCl for the EOR, both at a scan rate of 5 mV s^–1^, in an aqueous H_2_SO_4_ 0.5 mol L^–1^ solution with and without 1 mol L^–1^ absolute ethanol.

In order to study the electrocatalytic stability, chronoamperometry experiments were performed using H_2_SO_4_ 0.5 mol L^–1^ + 1 mol L^–1^ absolute ethanol over 3600 s, at 500 mV versus SHE for the anodic experiment and −26 mV versus SHE for the cathodic experiment.

Electrochemical impedance spectroscopies (EIS) were performed at different potentials in the frequency range from 100 kHz to 0.10 Hz (10 frequencies per decade) with a sinusoidal amplitude wave of 5 mV A.

All voltammograms presented in this study were represented versus SHE.

### 2.3 Hydrogen Production in Full Cell Configuration (Pt/SnO_2_/Nb_2_O_5_/Nb || Pt/SnO_2_/Nb_2_O_5_/Nb)

The membrane-free total electrolysis full cell configuration was built to evaluate ethanol selective oxidation, coupling with hydrogen production. Using a two-electrode configuration, the electrocatalyst Pt/SnO_2_/Nb_2_O_5_/Nb was used as the anode and cathode (Pt/SnO_2_/Nb_2_O_5_/Nb||Pt/SnO_2_/Nb_2_O_5_/Nb), with the distance between electrodes up to 1 cm. Electrochemical reforming was performed in an aqueous mixture of H_2_SO_4_ 0.5 mol L^–1^ + 1 mol L^–1^ absolute ethanol. To obtain the polarization curve of the cell (applied voltage vs current density), a constant polarization experiment was carried out at different potentials from 0.3 to 1.0 V, scaled by 0.1 V. The Faradaic efficiency (FE) of the cell was determined using the same gasovolummeter and general procedure described by Prieto et al.^6^ The FE (expressed in percentage) was determined according to the following equation

$$FE=\frac{V_{H_{2},\exp}}{V_{H_{2},the}}\times 100$$

where *V*_H2_,_exp_ is the experimental hydrogen produced during a chronoamperometric experiment at a constant voltage of 1.1 V over 2 h, and *V*_H2,the_ is the theoretical volume of hydrogen produced during the experiment (see S1 for more details). To determine the purity of the produced hydrogen, a chromatography analysis of the gases was performed using Shimadzu GC-14B Gas Chromatograph equipment (with a Carboxen column, thermal conductivity detector, and Ar as a carrier gas).

## 3 Results and Discussion

### 3.1 Physicochemical and Electrochemical Characterization

The FEG-SEM micrographs in Figure 1 show the surface morphologies of the electrocatalyst Pt/SnO_2_/Nb_2_O_5_/Nb (Figure 1a–d).

**Figure 1:**
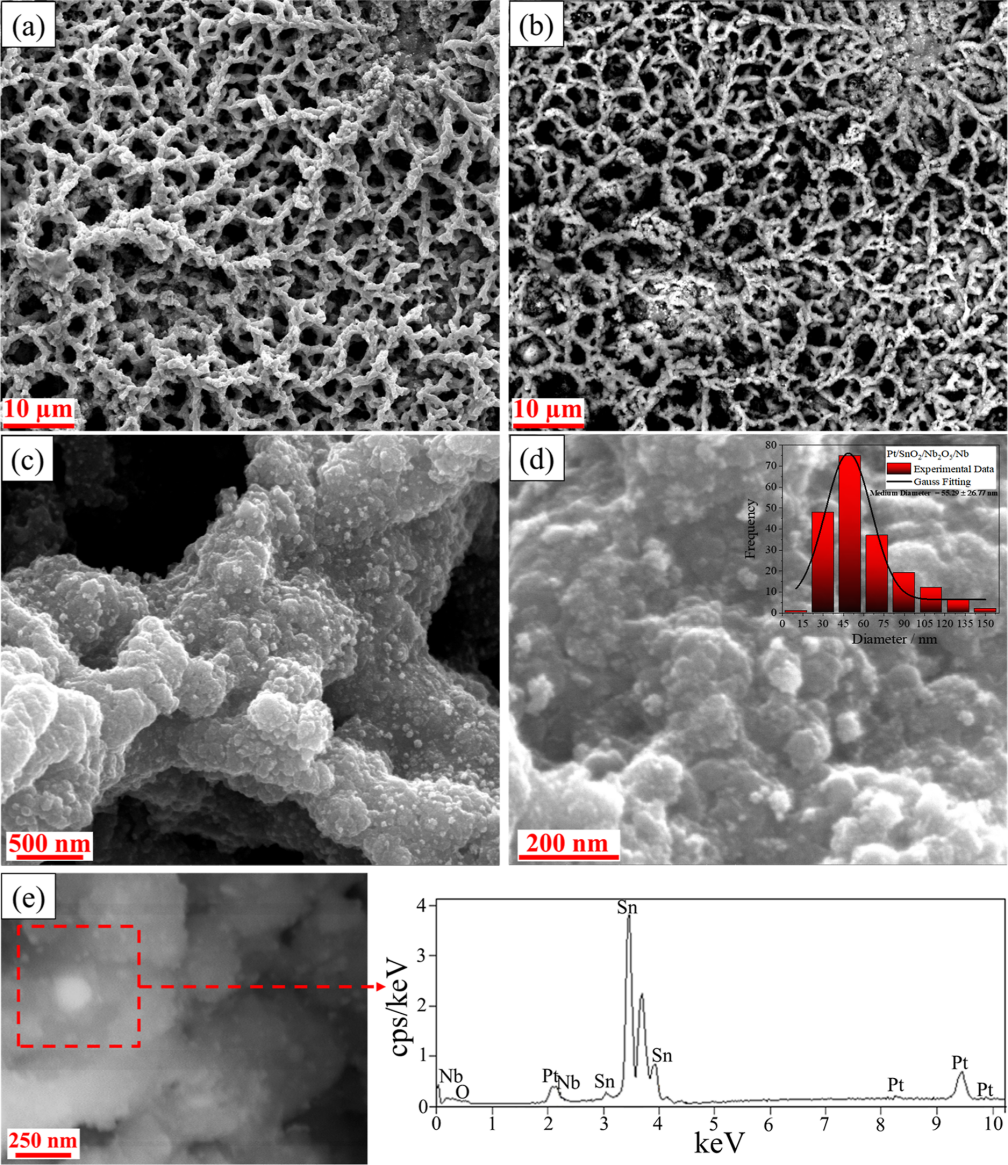
FEG-SEM morphology of the sample. (a–d): Pt/SnO_2_/Nb_2_O_5_/Nb with different magnifications. (e) EDS analysis area and obtained spectrum. Notes: Image (b) was obtained using backscattered electrons with the vCD detector; inset in (d): nanoparticle size distribution graph evaluated from a population of at least 200 nanoparticles.

The samples exhibit a rough and porous coral-like structure with highly branched structures formed due to gas generation during the PEO process.^44,45^ This coral-like morphology is advantageous for catalyst applications, providing better accessibility to a high number of active sites for reactions due to its large surface area, as shown in the literature.^46,47^

The FEG-SEM image obtained with a backscattered electron detector (vCD) (Figure 1b) allowed the detection of differences in the atomic densities of the elements, where the lighter color represents heavier elements. This lower-magnification image is representative and demonstrates that the nanoparticles are uniformly deposited on the coral-like cocatalyst surface, indicating that all areas are covered with Pt. The EDS results presented in Figure 1e confirm the presence of platinum in a more specific region of the sample surface at higher magnification, showing that the spherical-morphology structures observed in the backscattered image correspond to platinum nanoparticles formed on the sample after the solvothermal process.

The platinum-decorated material exhibits a range of particle sizes, as shown in the nanoparticle size distribution graph presented in the inset of Figure 1d, with an average nanoparticle size of 55.29 ± 26.77 nm. This distribution, combined with the small particle size, can contribute to a high density of active sites, enhancing the material’s performance in electrocatalytic reactions.

The structural characterization of the synthesized catalysts was carried out using grazing incidence X-ray diffraction (GIXRD). Figure 2 shows the diffraction patterns obtained for PEO samples Nb_2_O_5_/Nb (Figure 2a), SnO_2_/Nb_2_O_5_/Nb (Figure 2b), and Pt/SnO_2_/Nb_2_O_5_/Nb (Figure 2c).

**Figure 2:**
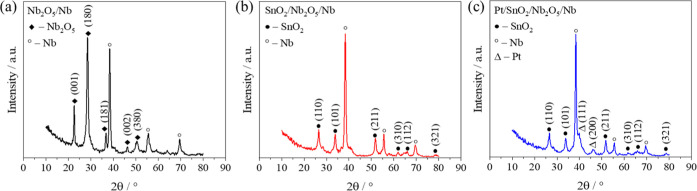
GIXRD patterns of Nb_2_O_5_/Nb (a), SnO_2_/Nb_2_O_5_/Nb (b), and Pt/SnO_2_/Nb_2_O_5_/Nb (c) samples. Reprinted from ref 38 with permission from Elsevier, Copyright 2025.

For understanding the structure of the Nb_2_O_5_ layer formed from the metallic niobium substrate during the PEO process, the Nb_2_O_5_ sample is synthesized without adding a tin precursor to the PEO electrolyte. The peaks at 2θ = 38.3°, 55.6°, and 69.5° are observed in all samples and can be associated with metallic niobium.^48–50^ The characteristic peaks of niobium pentoxide (Nb_2_O_5_) are identified at 2θ = 22.5°, 28.5°, 36.6°, 46.2°, 50.7°, and 55.5° can be, respectively, indexed to (001), (180), (181), (002), (380), and (182), assigned to an orthorhombic phase of Nb_2_O_5_ (JCPDS card no. 27-1003).^51,52^

In the SnO_2_/Nb_2_O_5_/Nb sample (Figure 2b), the characteristic peaks of SnO_2_ were observed at 2θ = 26.6°, 33.9°, 51.8°, 55.5°, 61.8°, 66.1°, and 78.7°. These peaks can be labeled with Miller indices (110), (101), (211), (220), (310), (112), and (321), respectively, which correspond to the tetragonal structure of the tin oxide (SnO_2_) cassiterite polycrystalline phase (JCPDS 77-0452).^45,53^

After the solvothermal platinum decoration process, the Pt/SnO_2_/Nb_2_O_5_/Nb sample (Figure 2c) showed the same GIXRD spectrum as the SnO_2_/Nb_2_O_5_/Nb sample, with two characteristic platinum peaks at 2θ = 40.0° and 46.6°, corresponding to the (111) and (200) planes of the face-centered cubic (FCC) Pt structure, respectively (JCPDS card no. 04-1802).^54^

In the GIXRD spectra obtained after each deposition step, no significant changes in the existing peaks were observed, indicating that no drastic structural modifications occurred during the process. The addition of new peaks corresponding to the newly incorporated elements is clearly evident. In the case of SnO_2_ deposition on Nb_2_O_5_, the Nb_2_O_5_ peaks disappeared, which can be attributed to the thickening of the SnO_2_ layer that covered the Nb_2_O_5_ peaks due to the higher amount of material deposited. After the addition of Pt to SnO_2_, the SnO_2_ peaks remained unchanged, while the characteristic Pt peaks appeared, confirming the deposition of platinum onto the material.

The platinum content in the electrocatalysts was quantified by ICP-OES. The measured average platinum loading was 0.052 ± 0.012 mg.

The concentrations of Pt and Nb_2_O_5_ in the Pt/SnO_2_/Nb_2_O_5_/Nb material depend on the operational parameters. The obtained contents are directly correlated with the conditions applied in the synthesis processes, including both the PEO step and the solvothermal deposition.

The Pt content in the catalyst directly impacts catalytic activity in both the HER and EOR, as platinum serves as the main active site for these reactions. Excessive Pt loading can lead to particle agglomeration, reducing the available active surface area and consequently decreasing catalytic efficiency.^55,56^ The presence of Nb_2_O_5_ is a consequence of the PEO synthesis process, in which the oxide forms from the niobium sheet and there is no direct effect on the HER and EOR; however, the coral-like structure of the SnO_2_ cocatalyst grows on Nb_2_O_5_ as part of the process.

The linear voltammograms obtained in the anodic sweep direction (which leads to the EOR) are shown in Figure 3a. In the presence of ethanol in a H_2_SO_4_ medium, the Nb_2_O_5_/Nb and SnO_2_/Nb_2_O_5_/Nb samples showed no increase in current density within the analyzed potential range, remaining at the same level as that in H_2_SO_4_ alone. This indicates that both samples are inactive for ethanol electrooxidation.

**Figure 3:**
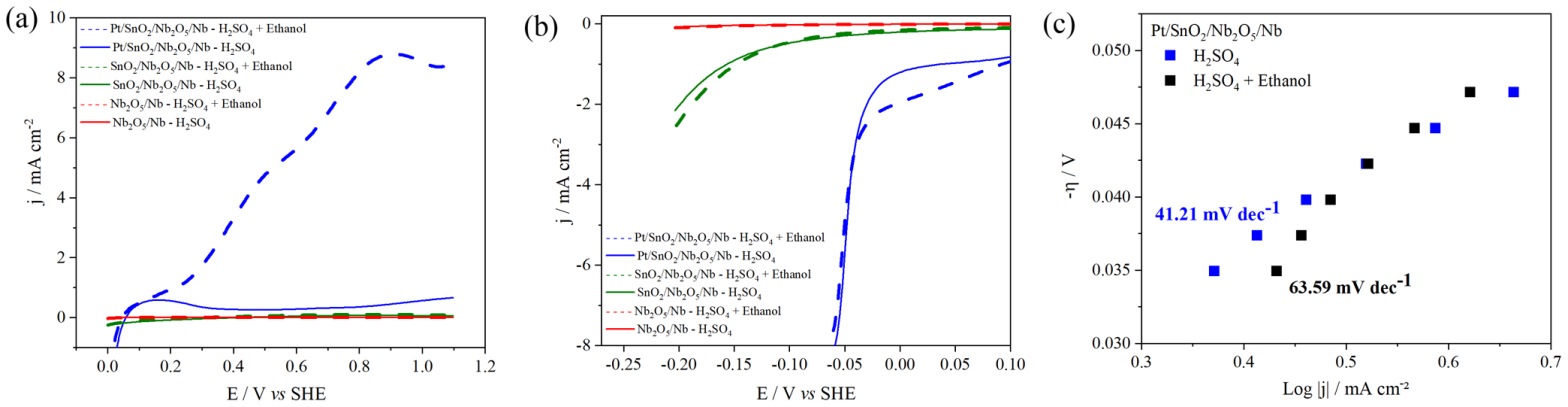
Linear voltammograms of (a) anodic sweep sense in 0.5 mol L^–1^ H_2_SO_4_ with and without 1.0 mol L^–1^ ethanol, (b) cathodic sweep sense for the HER in 0.5 mol L^–1^ H_2_SO_4_ with and without 1.0 mol L^–1^ ethanol, obtained at 5 mV s^–1^. (c) Tafel plots obtained from the voltammograms of the Pt/SnO_2_/Nb_2_O_5_/Nb sample.

The Pt/SnO_2_/Nb_2_O_5_/Nb sample showed, in the absence of ethanol (blue curve), a broad peak between 0 V and 350 mV versus SHE that demonstrates the typical profile of hydrogen absorption/desorption on Pt surfaces in an acid medium.^57^ After 350 mV, the current remains steady, indicating capacitive currents resulting from the accommodation of ions and dipoles in the double layer. Consequently, no significant faradaic reactions are occurring within this range.^27^

In the presence of ethanol (black curve), after 130 mV versus SHE, the Pt/SnO_2_/Nb_2_O_5_/Nb sample showed an increase in the current density, indicating the start of the EOR (*E*_onset_). This obtained value is slightly larger than the theoretical *E*_onset_ for the EOR (+84 mV vs SHE), but it is still much lower than the theoretical *E*_onset_ necessary for EWS (+1.23 V vs SHE).

During linear voltammetry, oxidation reactions of acetaldehyde and acetic acid also occur, as previously demonstrated for this material through in situ Fourier-transform infrared (ATR-FTIR) spectroscopy analyses.^38^ This discrepancy of 46 mV between the theoretical and observed values for EOR *E*_onset_ (black curve) may be attributed to these additional reactions and material-specific factors.

The linear voltammetry measurements in an acidic medium with 1 mol L^–1^ ethanol revealed a peak current density of 8.97 mA cm^–2^. The polarization curve exhibited two peaks: the initial peak developed from the full oxidation of ethanol to CO_2_ through the acetaldehyde and acetic acid route, while the subsequent peak originated from the partial oxidation of ethanol to acetaldehyde and acetic acid.

In the Pt/SnO_2_/Nb_2_O_5_/Nb system, both SnO_2_ and Nb_2_O_5_ act as supports and cocatalysts, enabling the electrode, produced from the metallic niobium substrate via PEO, to be self-supported. Additionally, SnO_2_ contributes to CO poisoning resistance by facilitating the oxidation of adsorbed intermediates during the EOR, thereby preserving platinum activity over time. These effects were previously discussed in our earlier study, where the electrocatalyst synthesis and EOR mechanisms were introduced.^38^

The linear voltammograms of the cathodic sweep direction (which leads to the HER) for the coral-like electrocatalyst are illustrated in Figure 3b. Once again, the Nb_2_O_5_/Nb and SnO_2_/Nb_2_O_5_/Nb samples exhibited no significant activity for the reaction of interest.

The Pt/SnO_2_/Nb_2_O_5_/Nb curves exhibit two distinct regions; at higher positive potentials (0 V–0.1 V vs SHE), a region of low and relatively constant current density is attributed to the formation of an electrochemical double layer due to H^+^ electro-adsorption.^6^ After the onset potentials (*E*_onset_) of −14 mV (H_2_SO_4_ 0.5 mol L^–1^ solution) and −26 mV (H_2_SO_4_ 0.5 mol L^–1^ + 1 mol L^–1^ ethanol solution), the current sharply increases toward negative values. This indicates the initiation of the HER in the electrocatalyst.^6^

In the HER, the coral-like Pt/SnO_2_/Nb_2_O_5_/Nb electrocatalyst shows a spontaneous and fast current increase, with an overpotential at −48 mV for a current density of 5 mA cm^–2^. This may be associated with the surface distribution and particle size of the Pt content.^58^ In addition, the porous morphology of the coral-like structure can facilitate the electrolyte diffusion on the surface, increasing contact between the solution and the active sites of the catalyst, where the HER occurs.

Figure 3c shows the Tafel plots obtained for the Pt/SnO_2_/Nb_2_O_5_/Nb sample. The Tafel slope value is 41.21 mV dec^–1^ for the solution without ethanol, which agrees with the other values obtained for Pt catalysts in the H_2_SO_4_ medium.^27,59^ When ethanol is added to the acid medium, the Tafel slope acquires a slightly higher value (63.58 mV dec^–1^). This suggests that the presence of ethanol in the medium may have slightly decreased the HER kinetics since lower values of the Tafel slope indicate faster HER kinetics.^6^

The Tafel slope also indicates the HER mechanism, which is usually divided into three. Initially, protons from the electrolyte are adsorbed onto the surface of the electrocatalyst, resulting in the formation of hydrogen atoms adsorbed at the active site, a process known as the Volmer step (120 mV dec^–1^). Subsequently, these adsorbed hydrogen atoms can either interact with another proton, accepting electrons to generate H_2_, known as the Heyrovsky step (40 mV dec^–1^), or two neighboring hydrogen atoms can combine to form molecular hydrogen, referred to as the Tafel step (30 mV dec^–1^).^60,61^ The mechanism of the HER for the Pt/SnO_2_/Nb_2_O_5_/Nb electrocatalyst is attributed to the Volmer–Heyrovsky step.

The Nyquist plot obtained from electrochemical impedance spectroscopy (EIS) in the absence of ethanol is shown in Figure 4a.

**Figure 4:**
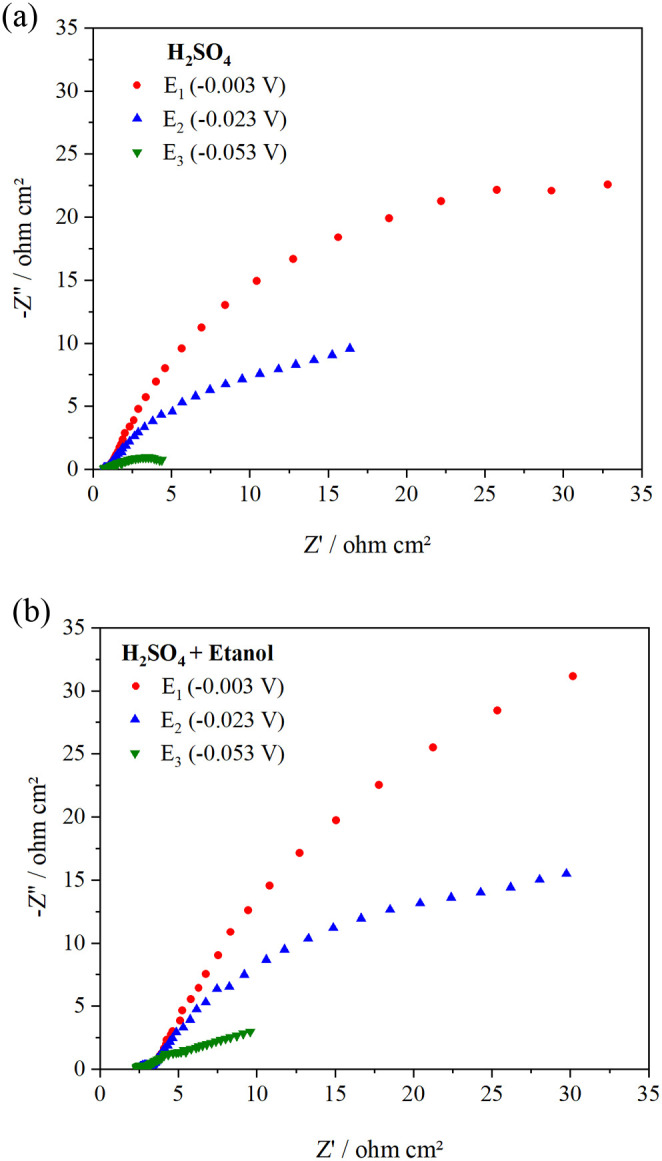
Nyquist plots obtained for (a) 0.5 mol of L^–1^ H_2_SO_4_ and (b) 0.5 mol of L^–1^ H_2_SO_4_ + 1.0 mol of L^–1^ ethanol at different cathodic potentials.

At high frequencies, the sample presents a potential-dependent semicircle (see Figure S4 in the Supporting Information), which can be related to the electrochemical charge transfer resistance across the particles and the particle–current collector. A second semicircle can be observed at lower frequencies, with a potential-dependent diameter. The smallest arc diameter can be observed in the applied potential of −0.053 V (E_3_), determining a RCT value of ≈5.0 Ω cm^2^. As discussed for the voltammogram in Figure 3b, close to this potential, the HER begins to occur. So, this RCT can be associated with the electrochemical charge transfer resistance of the HER (*R*_CT_) on the studied electrocatalyst.^6^ This RCT value obtained for the HER overpotential is bigger than other Pt-based electrocatalysts reported in the literature.^36,62^ The Nyquist plot with ethanol is shown in Figure 4b. In this case, the semicircles show the same behavior as in the absence of ethanol, but they present a slight increase in *R*_CP_ values. This indicates that the presence of ethanol in the acid medium affects the electrochemical behavior of the HER, as demonstrated in Figure 3.

This study uses the Nyquist plot primarily for a qualitative comparison of the systems, complementing the electrochemical findings. The focus of the analysis is on charge transfer resistance without incorporating an equivalent circuit at this time.

The Pt/Nb_2_O_5_ system without SnO_2_, when subjected to this solvothermal deposition method, showed no deposition of Pt nanoparticles on the Nb_2_O_5_ coating by this method, resulting in the absence of significant electrocatalytic activity. Therefore, experiments with this system were not conducted.

### 3.2 Stability Tests of the Electrocatalyst

Chronoamperograms acquired at a constant potential corresponding to anodic and cathodic overpotentials are shown in Figure 5. Both plots exhibit a characteristic pattern featuring an exponential decrease in current initially, attributed to capacitive currents, and then show a slow decay predicted for this type of electrochemical system according to the Cottrell equation.^63^ Subsequently, this demonstrates a gradual decline expected in such electrochemical systems. The Pt/SnO_2_/Nb_2_O_5_/Nb electrocatalyst presents good activity retention and stability for the EOR and HER, maintaining constant current for 12 h of polarization, with no distortion of current caused by the release of H_2_ bubbles during the cathodic experiment.

**Figure 5:**
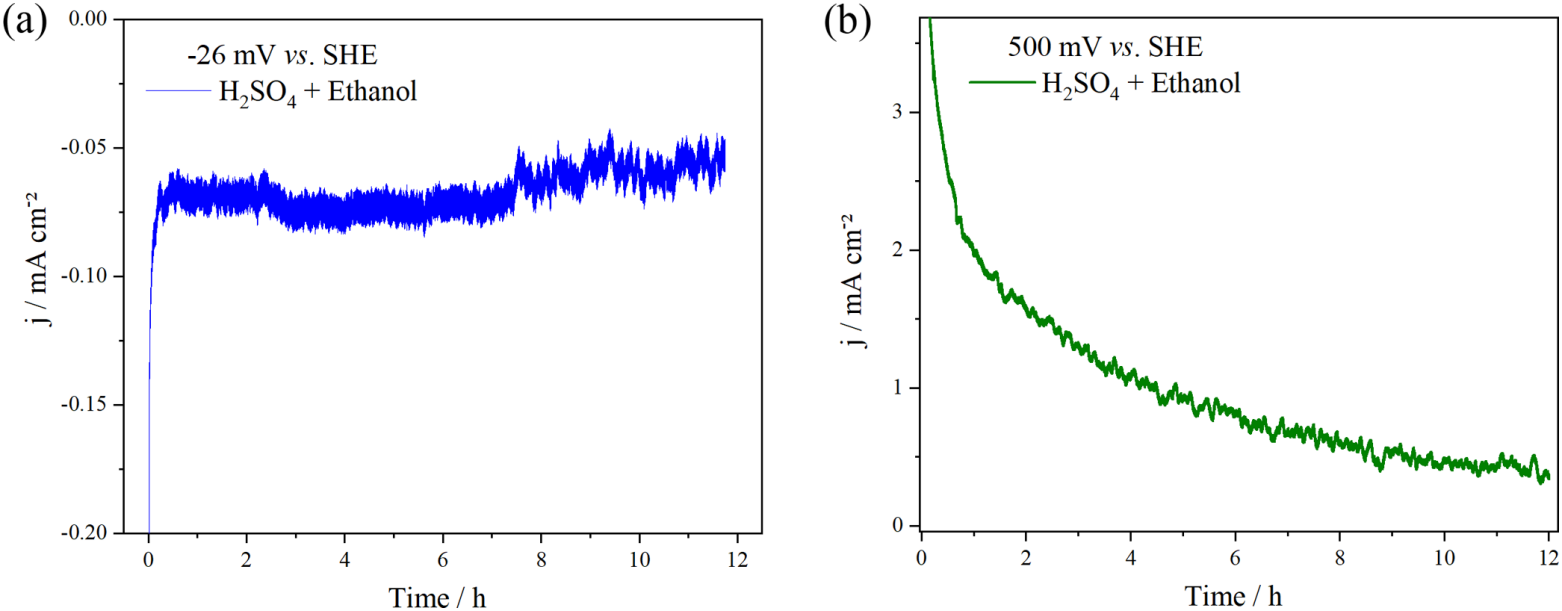
Chronoamperograms of the Pt/SnO_2_/Nb_2_O_5_/Nb sample in 0.5 mol L^–1^ H_2_SO_4_ with 1.0 mol L^–1^ ethanol solution at 25 °C. (a) Cathodic potential (−26 mV vs SHE) and (b) anodic potential (500 mV vs SHE).

After the stability tests, the samples of the Pt/SnO_2_/Nb_2_O_5_/Nb system were subjected to XRD characterizations (Figure 6).

**Figure 6:**
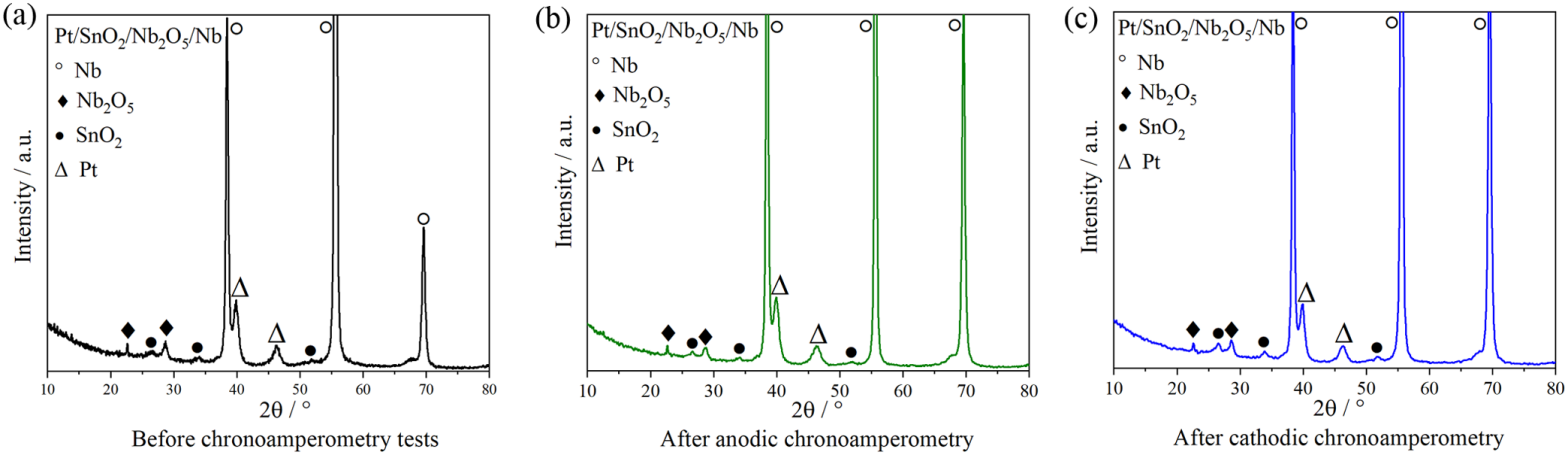
XRD diffraction patterns of the Pt/SnO_2_/Nb_2_O_5_/Nb sample. (a) Before chronoamperometry tests, (b) after the anodic chronoamperometry test, and (c) after the cathodic chronoamperometry test.

It is observed that the diffraction peaks did not change when comparing the samples before and after the chronoamperometry tests (anodic and cathodic), with peaks related to SnO_2_, Nb_2_O_5_, and Nb clearly identified, as previously discussed (Figure 2). Additionally, the peaks associated with the crystalline phases of platinum remained unchanged, demonstrating the physicochemical stability of the catalyst after the stability test.

Unlike the graphs presented in Figure 2, the graphs obtained after the chronoamperometry tests were not acquired with grazing incidence X-ray. As a result, the X-ray penetration into the sample was significantly higher, leading to more intense peaks associated with the metallic niobium substrate, compared to those presented previously.

After the stability tests, the electrolytes were analyzed by ICP-OES to investigate the possible leaching of ions from the electrocatalyst into the medium. The analysis did not detect the presence of Nb or Sn. The amount of platinum found in the electrolyte corresponded to approximately 3% of the total Pt mass initially present in the Pt/SnO_2_/Nb_2_O_5_/Nb electrocatalyst.

### 3.3 Hydrogen Production in Full Cell Configuration

The Pt/SnO_2_/Nb_2_O_5_/Nb material can act as a bifunctional electrocatalyst for both the HER and EOR with remarkable activity and stability. In this way, H_2_ generation from the EOR (Figure 7, Pt/SnO_2_/Nb_2_O_5_/Nb||Pt/SnO_2_/Nb_2_O_5_/Nb inset of; Video S1, Supporting Information) is effectively boosted since the sluggish OER on the anode will be replaced by the fast ethanol electrooxidation on the surface of the catalyst.

**Figure 7:**
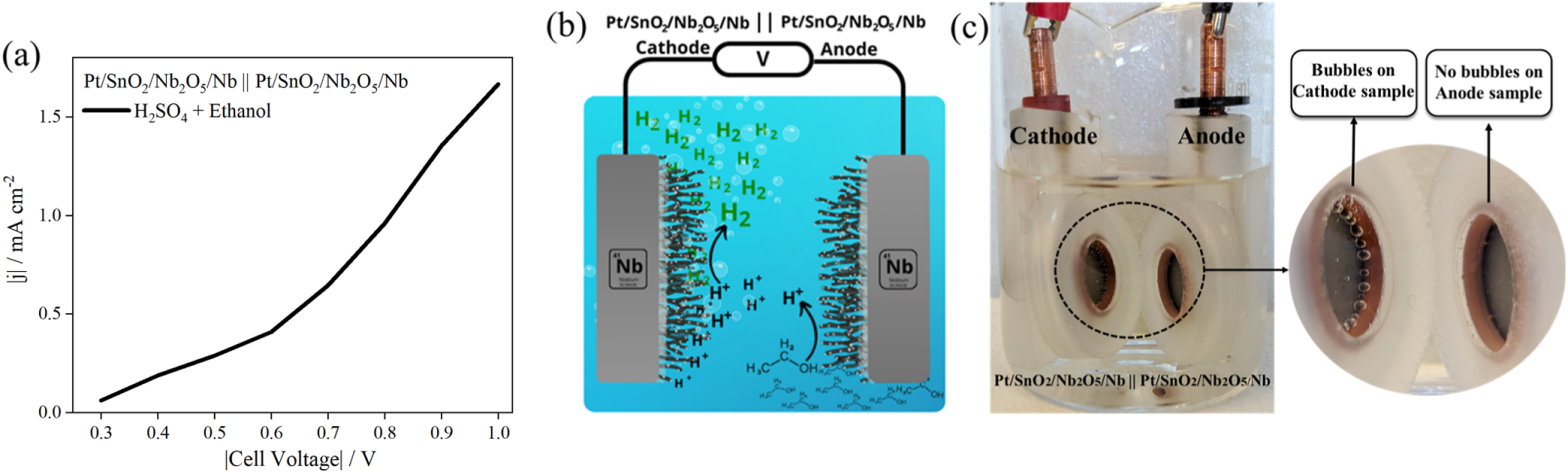
(a) Polarization curves of Pt/SnO_2_/Nb_2_O_5_/Nb||Pt/SnO_2_/Nb_2_O_5_/Nb for hybrid water splitting. The potential and current values are represented in absolute magnitude. (b) Schematic illustration of the bifunctional electrocatalysts for both the EOR and HER in a two-electrode system. (c) Visual representation of the cell mounted on two electrodes, generating bubbles only at the cathode; photo taken by the author.

As a bifunctional apparatus (Pt/SnO_2_/Nb_2_O_5_/Nb||Pt/SnO_2_/Nb_2_O_5_/Nb), the device facilitates ethanol electrolysis at the anode, releasing H_2_ at the cathode. Based on the results of linear voltammetry of three-electrode cell configuration (Figure 3a,b), the *E*_onset_ for the EOR is 130 mV versus SHE and the *E*_onset_ for the HER is −26 mV versus SHE. Thus, hypothetically, the full cell potential required to generate H_2_ from the EOR is 156 mV vs SHE. Observing Figure 7a, there is an increase in current density of up to 600 mV, coinciding with the beginning of bubble detachment from the cathode, as illustrated in Video S1 (Supporting Information). Pt/SnO_2_/Nb_2_O_5_/Nb is depicted in Figure 7b,c as a bifunctional electrocatalyst for the EOR and HER when utilized in a full cell configuration.

The gaseous products from the Pt/SnO_2_/Nb_2_O_5_/Nb||Pt/SnO_2_/Nb_2_O_5_/Nb bifunctional electrocatalyst are quantified by gas chromatography. Only H_2_ and air are detected (Figure S2, Supporting Information). It is important to point out that no other peaks related to CO or CO_2_ gases were registered in the chromatograph. This agrees with the spectroelectrochemical results, where CO and CO_2_ gases were not detected.^38^

Our primary hydrogen quantification was based on a gas-volumetric system (Figure S1, Supporting Information) to measure the gas volume after the electrolysis process, following previous studies.^6^ Additionally, gas chromatography analysis confirmed that the measured volume corresponds solely to hydrogen. In future studies, we intend to implement gas chromatography to monitor hydrogen production over time, as done by other authors.^64^

An important aspect in the evaluation of materials for electrolyzers is the determination of the FE. This parameter indicates what portion of the electrical energy used in the process is used for the intended purpose, in this case, hydrogen production. According to the obtained results (Supporting Information), the calculated FE is 99.7 ± 3.1%. Considering the uncertainty of this value, mainly affected by the uncertainty of the experimental determination of the produced hydrogen, it is possible to affirm that the FE of the cell is very close to 100%. This value indicates excellent efficacy of the bifunctional electrocatalyst for the use of electrical energy for the anodic (EOR) and cathodic (HER) cell reactions with very low parasitic currents.

Kweon et al.^65^ determined the FE of 85.97% with 1.8 V for the HER in a H_2_SO_4_ medium for the Pt/C electrode in water splitting experiments. Zhou et al.^24^ investigated the power consumption of hydrogen production by electrochemical reforming of ethanol in an alkaline medium. Using a two-electrode system with the Pt_3_Ni/NiF cathode, the FE of hydrogen production reached 94%.

Acetaldehyde, acetic acid, and CO_2_ are byproducts formed at the anode due to the EOR in the full cell configuration. According to the following reactions:^38,66^

Partial oxidation of ethanol to acetaldehyde (CH_3_CHO)

$${CH}_{3}{CH}_{2}OH\to {CH}_{3}CHO+2H^{+}+2e^{-}$$

Oxidation of acetaldehyde to acetic acid (CH_3_COOH)

$${CH}_{3}CHO+H_{2}O\to {CH}_{3}COOH+2H^{+}+2e^{-}$$

Complete oxidation of ethanol to carbon dioxide (CO_2_)­

$${CH}_{3}{CH}_{2}OH+3H_{2}O\to 2{CO}_{2}+12H^{+}+12e^{-}$$

Simultaneously, the HER takes place at the cathode, following the Volmer–Heyrovsky step mechanism (Figure 3c).

During the Volmer stage, a hydrogen ion (H^+^) in the solution is adsorbed onto the electrode surface, gains an electron, and becomes an adsorbed hydrogen atom (H*).^60,67^ This process is represented by the following chemical reaction

$$H^{+}+e^{-}\to H^{*}$$

In the Heyrovsky Step, the adsorbed hydrogen atom (H*) combines with a proton from the solution and an electron to produce molecular hydrogen gas (H_2_), which then detaches from the electrode surface. According to the following reaction

$$H^{*}+H^{+}+e^{-}\to H_{2}$$

Although the production of acetic acid may increase the acidity of the medium, the experiments were conducted in a 0.5 M H_2_SO_4_ solution, which has a low and stable pH and does not significantly affect the reaction efficiency. In commercial devices, the solution is constantly homogenized, and the ionization constant of acetic acid is weak, limiting its influence on the pH. Furthermore, only the local pH at the anode is altered during the oxidation reactions, which does not impact the H_2_ production at the cathode.

These findings prove that ethanol electrolysis with this coral-like electrocatalyst demands less energy input than water-splitting reactions, thus avoiding the formation of oxygen, eliminating the need for a PEM in the system, and generating high-value-added byproducts, such as acetic acid and acetaldehyde, during the reaction.

## 4 Conclusions

This study highlights the potential of Pt-decorated ceramic SnO_2_ coral-like structures, synthesized by PEO, as a bifunctional self-supported electrocatalyst for the EOR and HER in an acidic medium. The Pt/SnO_2_/Nb_2_O_5_/Nb catalyst exhibited efficacy for the EOR, with an *E*_onset_ value of +130 mV versus SHE and a peak current density of 8.97 mA cm^–2^. Furthermore, the bifunctional electrocatalyst demonstrated a spontaneous and rapid increase in current for the HER, achieving an overpotential at −48 mV for a current density of 5 mA cm^–2^ and a FE of 99.7 ± 3.1%. Chronoamperometry demonstrated that the Pt/SnO_2_/Nb_2_O_5_/Nb sample presents good activity retention and stability for the EOR and HER. The mechanism of the HER for the electrocatalyst is attributed to the Volmer–Heyrovsky step. This efficient coupling of the EOR with the HER significantly reduces energy consumption for green H_2_ generation compared to traditional water splitting. With H_2_ being the only gaseous product, the EOR–HER electrolysis system could operate in the absence of a PEM, enhancing the economic viability of the process and offering a sustainable pathway for green hydrogen production with valuable byproducts.

## Supplementary Material





## Notes

(MP4)
Faradaic efficiency (FE) determination for hydrogen production in full-cell configuration, photographs of the experimental setup, chromatogram graph, chronoamperometric curve for hydrogen production, and Nyquist plot in the high-frequency region for Pt/SnO_2_/Nb_2_O_5_/Nb (PDF)
