# Supplementary material for: A Self-Supported Coral-like Pt/SnO2/Nb2O5/Nb Electrode: A Bifunctional Electrocatalyst for Membrane-Free Green H2 Production via Coupling Ethanol Selective Electrooxidation
Source: ACS Omega. 2025 May 29;10(22):23297–307. doi: 10.1021/acsomega.5c01734 (PMC12163843; doi:10.1021/acsomega.5c01734)
Supplement: Supplementary file 2 [file ao5c01734_si_002.pdf]

Supporting Information

Self-supported coral-like $Pt/SnO_2/Nb_2O_5/Nb$ electrode: a bifunctional electrocatalyst for membrane-free green H_2 production via coupling ethanol selective electrooxidation

Matheus Bullmann [a], Andrés Cuña Suárez [b,c], Edna Jerusa Pacheco Sampaio [a], Natalia Prieto Pastorino [b], Roberto Hubler [d], Eva Chinarro [e], Célia de Fraga Malfatti [a]*

a Corrosion Research Laboratory (LAPEC), Engineering School, Federal University of Rio Grande do Sul (UFRGS), Av. Bento Gonçalves, 9500, 91501-970, Porto Alegre, Rio Grande do Sul, Brazil.

b Physical Chemistry Area, DETEMA, Faculty of Chemistry, University of the Republic (UDELAR), CC 1157, Montevideo 11800, Uruguay.

c Renewable Energies Laboratory, Pando Technological Pole Institute, Faculty of Chemistry, University of the Republic (UDELAR), Route 8 Km 17.500, 91000 Pando, Canelones, Uruguay.

d Polytechnic School, Pontifical Catholic University of Rio Grande do Sul (PUC-RS), Av. Ipiranga, 6681, 90619-900, Porto Alegre, Rio Grande do Sul, Brazil.

e I Institute of Ceramics and Glass (ICV), Higher Council for Scientific Research (CSIC), Cantoblanco Campus, C/ Kelsen 5, 28049 Madrid, Spain.

1. *Hydrogen production in full cell configuration: Determination of the Faradaic Efficiency (FE).*

The theoretical volume of the produced hydrogen ($V_{H2,the}$) was determined, using Faraday's Law and considering an ideal gas behavior, through Eq. 1 [1]:

$$V_{H_2,the} = \frac{Q_{H2} \times R \times T \times 1000}{n \times F \times P} \quad (1)$$

where $V_{H2,the}$ is the volume of the produced H_2 expressed in mL, Q_{H2} is the charge passing through the cell during the chronoamperometric experiment, R is the ideal gas constant (0.083144 L bar K^{-1} mol^{-1}), T is the temperature in K (291), η is the number of electrons exchanged during the HER (n = 2), F is the Faraday constant (96483.3 C mol^{-1}), P is the atmospheric pressure in bar (1.032 bar) and 1000 is the volume factor conversion from L to mL.

The experimental volume of the produced hydrogen ($V_{H2,exp}$) was measured in the gasovolummeter according to the experimental procedure used by Prieto et al[1]. The pictures in Figure S1 show the different parts of the experimental system. Finally, the FE can be obtained from the quotient between $V_{H2,exp}$ and $V_{H2,the}$, according to Eq. 2 [1]:

$$FE = \frac{V_{H_2,\,exp}}{V_{H_2,\,the}}\, x\, 100 \quad (2)$$

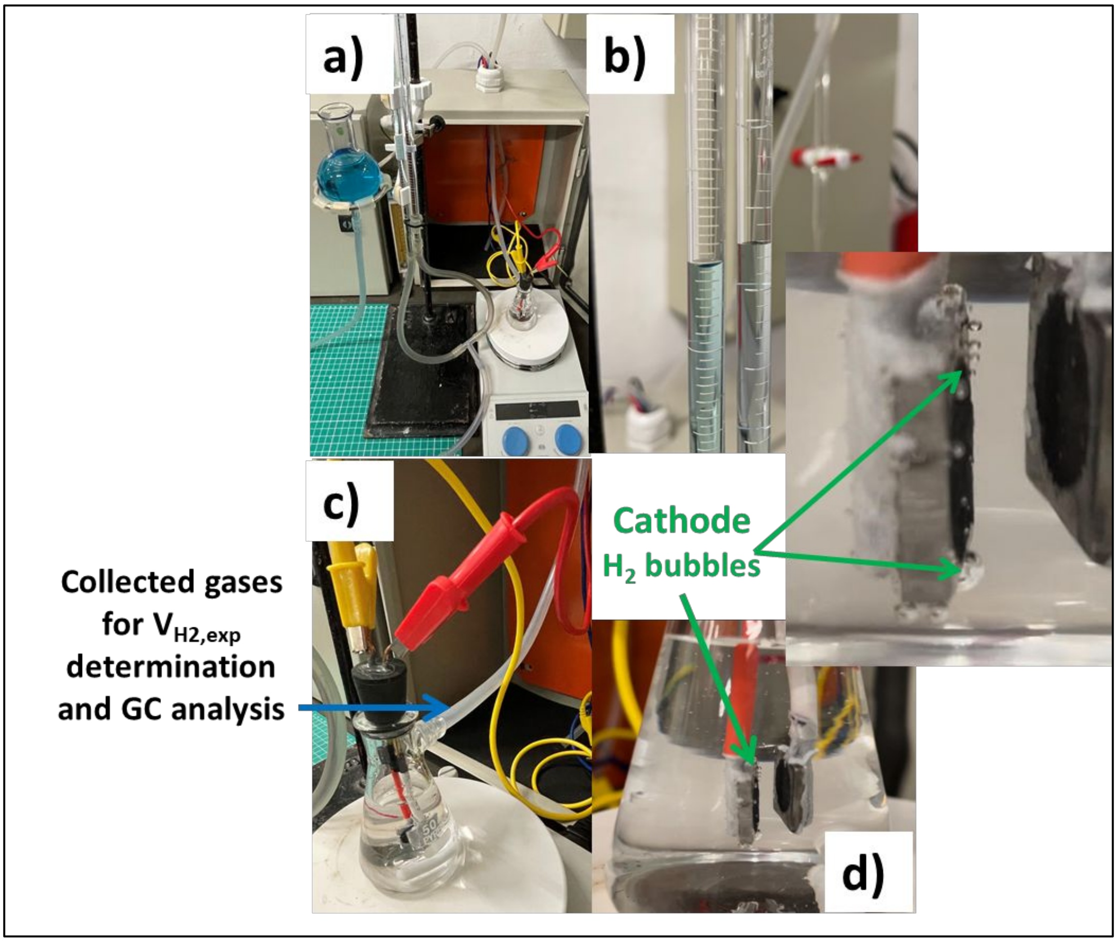

Figure S1. a) General view of the electrolytic cell connected to the gasovolummeter apparatus. b) Displacement of the columns in the gasovolummeter due to the volume of hydrogen generated during the experiment. c) Electrolytic cell showing the connection where the gas was collected for quantification and analysis. d) Close-up photo of the cathode and anode of the cell. The inset of the figure shows the formation of H_2 bubbles on the cathode surface.

The composition of the gas produced during the experiment was evaluated by a gas chromatography (GC) analysis. For that, 0.5 mL of the gases contained in the cell before and after the electrolytic experiment was collected and analyzed. Figure S2 shows both obtained chromatographs. The chromatograph of the gases collected after the electrolytic experiment (blue curve) clearly shows a peak at 1.15 min, corresponding to an H_2 gas,

while in the chromatograph obtained before the experiment (black curve) there is only one peak related to air (at 2.16 min). It is important to point out that no other peaks related to CO or CO_2 gases were registered in the chromatograph. This agrees with the spectroelectrochemical results where CO and CO_2 gases were not detected[2], and gives us the conclusion that these gases are not products of the EOR in the cell anode. So, we can assume that the gas formed during the electrolytic experiment for $V_{H2,exp}$ quantification corresponds only to an H_2 gas.

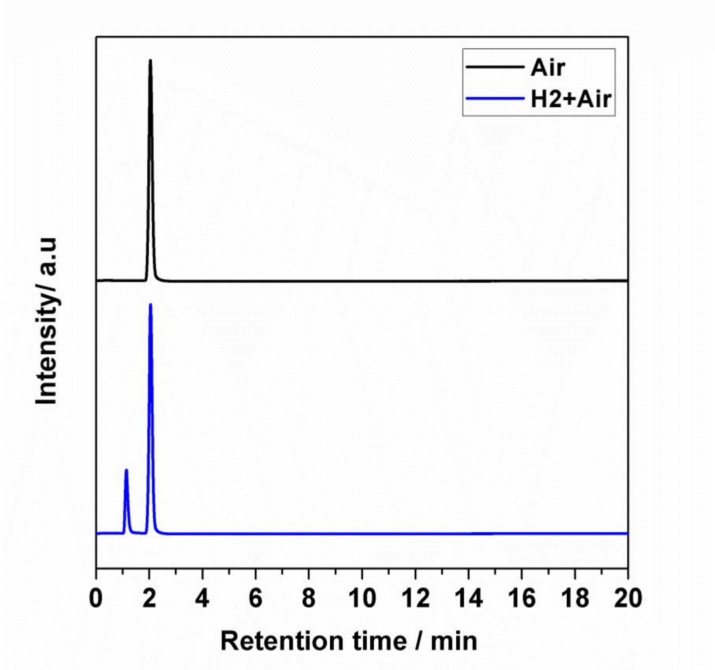

Figure S2. Chromatograph of the gases collected before (Air) and after the electrolytic experiment (H_2+Air).

The Figure S3 shows the I *vs.* time curve obtained from the chronoamperometric experiment using a PalmSens potentiostat/galvanostat equipment (model EmStat4S High Range). The total charge value (Q_{H2} = 2.68 C) is calculated from the integral of the curve and the $V_{H2,the}$ (= 0.326 mL) value is determined with Eq. 1. Considering the $V_{H2,exp}$ (=

0.325 mL) measured with the gasovolummeter apparatus, a FE of 99.7 % was calculated with Eq. 2.

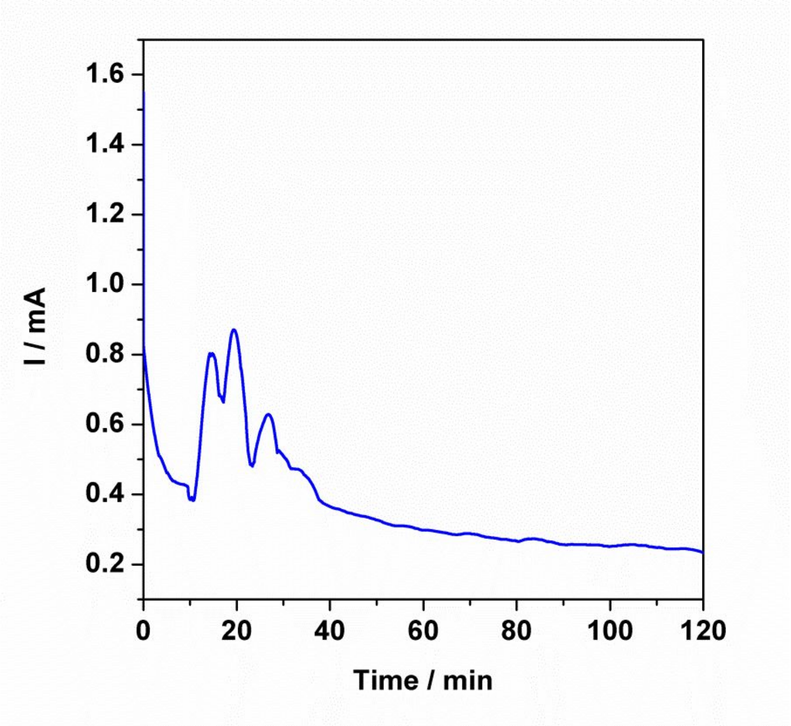

Figure S3. Chronoamperometric curve obtained at 1.1 V over 2 h at a constant potential of 1.1 V.

The Figure S4. provides a close-up of the Nyquist plot in the absence of ethanol, showcasing the first semicircle at high frequencies that is indicative of the electrochemical charge transfer resistance between the particles and the current collector.

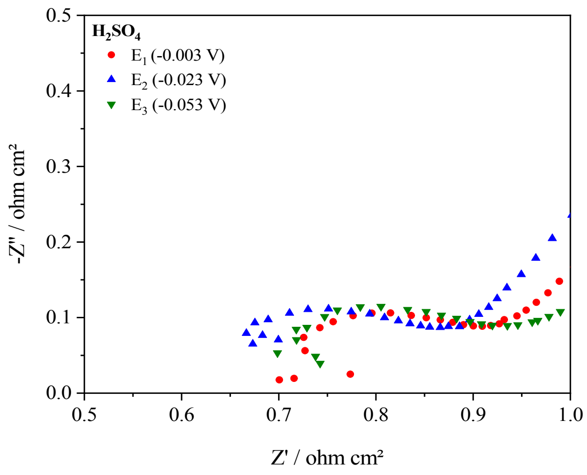

Figure S4. Nyquist plot in high frequences for $Pt/SnO_2/Nb_2O_5/Nb$ in 0.5 mol L^{-1} H_2SO_4 at different cathodic potentials.

References

[1] Prieto N, da Silva EL, Castiglioni JR, Cuña A. Synthesis and characterization of non-noble metal cathode electrocatalysts for PEM water electrolysis. Electrochimica Acta 2024;473:143474. https://doi.org/10.1016/j.electacta.2023.143474.

[2] Bullmann M, Etcheverry L, Suárez AC, Sampaio EJP, Pitthan E, Andrade AMH de et al. Tailored PEO synthesis and *in-situ* ATR-FTIR study of PtSnO2/Nb coral-like structures for application in ethanol electrooxidation. Journal of Alloys and Compounds 2024:175178. https://doi.org/10.1016/j.jallcom.2024.175178.
